# Effects of Presurgical Mandibular Incisor Decompensation on Long-Term Outcomes of Class III Surgical Orthodontic Treatment

**DOI:** 10.3390/jcm10132870

**Published:** 2021-06-28

**Authors:** Jung-Sub An, Wonchae Jeong, Liselotte Sonnesen, Seung-Hak Baek, Sug-Joon Ahn

**Affiliations:** 1 Department of Orthodontics, Seoul National University Dental Hospital, 101 Deahakno, Jongno-Gu, Seoul 03080, Korea; jungsub.an@gmail.com; 2 Dental Research Institute and Department of Orthodontics, School of Dentistry, Seoul National University, 101 Deahakno, Jongno-Gu, Seoul 03080, Korea; j2wch@naver.com (W.J.); drwhite@snu.ac.kr (S.-H.B.); 3 Section of Orthodontics, Department of Odontology, Faculty of Health and Medical Sciences, University of Copenhagen, 20 Nørre Alle, 2200 Copenhagen, Denmark; alson@sund.ku.dk

**Keywords:** Class III, surgical orthodontic treatment, orthognathic surgery, decompensation, presurgical orthodontic treatment

## Abstract

This research aimed to evaluate the effects of presurgical mandibular incisor decompensation on long-term outcomes of Class III surgical orthodontic treatment. Thirty-five patients with skeletal Class III malocclusion who received conventional surgical orthodontic treatment were included. Mandibular incisor brackets with −6° of inclination were placed normally in 18 patients (NB group) and inversely in 17 patients (RB group). Between-group differences and relationships between incisal and skeletal variables were analyzed based on lateral cephalograms at pretreatment, presurgery, postsurgery, posttreatment, and retention. Mandibular incisors were more labially inclined in the RB group than in the NB group from presurgery to retention. No significant between-group differences were observed in presurgical and postsurgical skeletal relationships. The NB group exhibited a larger overjet with deficient interincisal contact at postsurgery than the RB group. Skeletal Class III relationship was also more severe in the NB group at retention. More lingually inclined mandibular incisors at presurgery and larger overjet at postsurgery were correlated with a more severe skeletal Class III relationship at retention. Thus, establishing appropriate postsurgical overjet by sufficient presurgical mandibular incisor decompensation may play a significant role in postsurgical stability of Class III surgical orthodontic treatment.

## 1. Introduction

Patients with skeletal malocclusion generally exhibit dentoalveolar compensation to adapt their craniofacial skeletal patterns [1]. The most common dentoalveolar compensation in skeletal Class III malocclusion is retroclination of the mandibular incisors and proclination of the maxillary incisors [2], which help maintain occlusal function but may mask skeletal discrepancy [3]. Camouflage treatment using dentoalveolar compensation is a treatment option for patients with skeletal Class III malocclusion; however, when this approach is esthetically and functionally inappropriate, surgical orthodontic treatment should be considered [4].

Conventional surgical orthodontic treatment consists of three phases: (1) presurgical orthodontic treatment including decompensation, decrowding, and coordination of maxillomandibular arch; (2) orthognathic surgery; and (3) completion of the occlusion with postsurgical orthodontic treatment [5,6]. During presurgical orthodontic treatment of skeletal Class III malocclusion, elimination of dentoalveolar compensation including proclination of the mandibular incisors and retroclination of the maxillary incisors should be performed to ensure a desirable inclination of incisors to the basal bone and to expose the original skeletal discrepancy [4]. Such decompensation determines the degree of surgery and contributes to long-term stability by stabilizing the postsurgical occlusion [5,7,8,9]. However, as facial appearance and occlusal function of patients may deteriorate during presurgical decompensation, many patients opt to undergo surgery at an early stage to improve their appearance [6]. In this regard, minimal presurgical orthodontic treatment [10] and surgery-first [11] approaches have been introduced.

Insufficient decompensation during presurgical orthodontic treatment is a major contributor to inadequate improvement of skeletal discrepancy in surgical cases [4,12,13]. However, a consensus has yet to be reached regarding the optimal degree of presurgical decompensation required to obtain desired surgical outcomes and long-term stability. In particular, long-term assessments of dentoskeletal changes related to presurgical mandibular incisor decompensation in Class III patients are lacking. The purpose of this retrospective study was to evaluate the effects of presurgical mandibular incisor decompensation on long-term outcomes of Class III surgical orthodontic treatment. To this end, dentoskeletal changes were compared between patients with and those without sufficient presurgical decompensation of the mandibular incisor inclination at pretreatment, presurgery, postsurgery, posttreatment, and retention. The null hypothesis was that long-term treatment outcomes would not differ significantly between Class III surgical patients with and without sufficient presurgical decompensation of the mandibular incisor inclination.

## 2. Materials and Methods

### 2.1. Participants

Skeletal Class III patients who underwent surgical orthodontic treatment at the Seoul National University Dental Hospital between January 2010 and November 2017 were included in this retrospective study. Inclusion criteria were as follows: (1) skeletal Class III patients with ANB angle ≤0° at pretreatment; (2) minimum age of 17 years at pretreatment given that circumpubertal growth in Koreans is generally complete or almost complete at this age [14,15]; (3) bimaxillary orthognathic surgery, including Le Fort I and bilateral sagittal split ramus osteotomy; (4) conventional surgical orthodontic treatment using 0.022-inch brackets with MBT prescription [16] and a 0.019 × 0.025-inch stainless steel wire for surgical stabilization of the mandibular arch; (5) crowding or spacing <2.0 mm in the mandibular arch at pretreatment; (6) non-extraction in the mandibular arch excluding the third molars; and (7) complete series of lateral cephalograms at five stages including pretreatment (T1), presurgery (T2), postsurgery (T3), posttreatment (T4), and retention (T5; at least 1 year after orthognathic surgery). Patients with the following conditions were excluded: (1) craniofacial syndromes; (2) history of trauma; (3) additional maxillofacial surgery excluding genioplasty; (4) history of temporomandibular disorder; (5) missing teeth; and (6) prosthodontic treatment of the maxillary and/or mandibular anterior teeth. In total, 35 patients were included in the study based on the inclusion and exclusion criteria.

Patients were divided into two groups according to the manner in which brackets were placed relative to the mandibular incisors: (1) in the normal bracketing group (NB), mandibular incisor brackets (MBT prescription [16]) were placed in the normal way with inclination set to −6°; (2) in the reverse bracketing group (RB), brackets were placed inversely with inclination of the mandibular incisors set to +6° [17]. This approach was adopted because patients in RB presented with thick alveolar housing to accommodate sufficient proclination of the mandibular incisors, whereas patients in NB presented with thin alveolar housing [18].

Power analysis was conducted using G*Power 3.1 (Heinrich-Heine, Düsseldorf, Germany). Sample size calculation based on the results of a pilot study indicated that at least 16 participants per group would be required with an alpha error of 0.05 and a power of 0.8 to identify a true difference between groups. The sample size was considered adequate given that NB and RB comprised 18 and 17 patients, respectively.

### 2.2. Cephalometric Measurements

**Figure 1:**
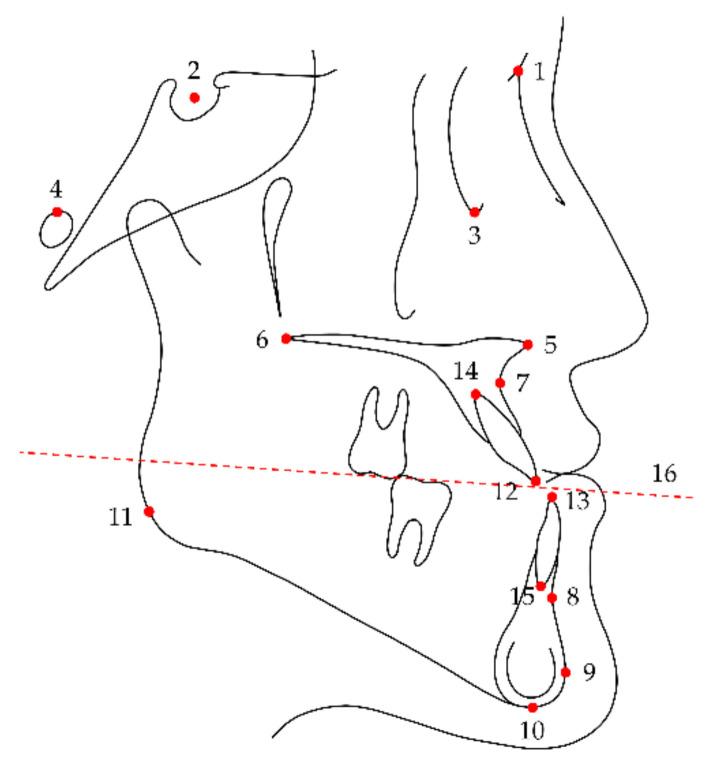
Cephalometric landmarks and reference plane used in this study: 1, nasion; 2, sella; 3, orbitale; 4, porion; 5, anterior nasal spine; 6, posterior nasal spine; 7, Point A; 8, Point B; 9, pogonion; 10, menton; 11, gonion; 12, incisal edge of the maxillary central incisor; 13, incisal edge of the mandibular central incisor; 14, root apex of the maxillary central incisor; 15, root apex of the mandibular central incisor; 16, occlusal plane (a line connecting the midpoint of the incisal edges of the maxillary and mandibular central incisors to the midpoint of the mesiobuccal cusps of the maxillary and mandibular first molars).

**Figure 2:**
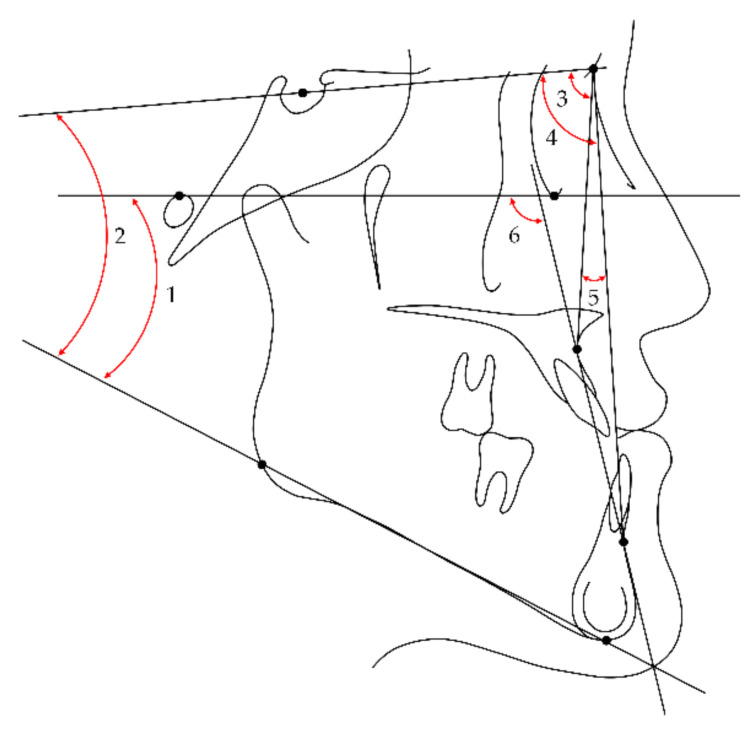
Skeletal cephalometric variables measured in this study (all variables were angular measurements): 1, Frankfort-mandibular plane angle (FMA); 2, sella-nasion to mandibular plane angle (SN-MP); 3, SNA; 4, SNB; 5, ANB; 6, Frankfort horizontal to AB plane angle (FABA).

**Figure 3:**
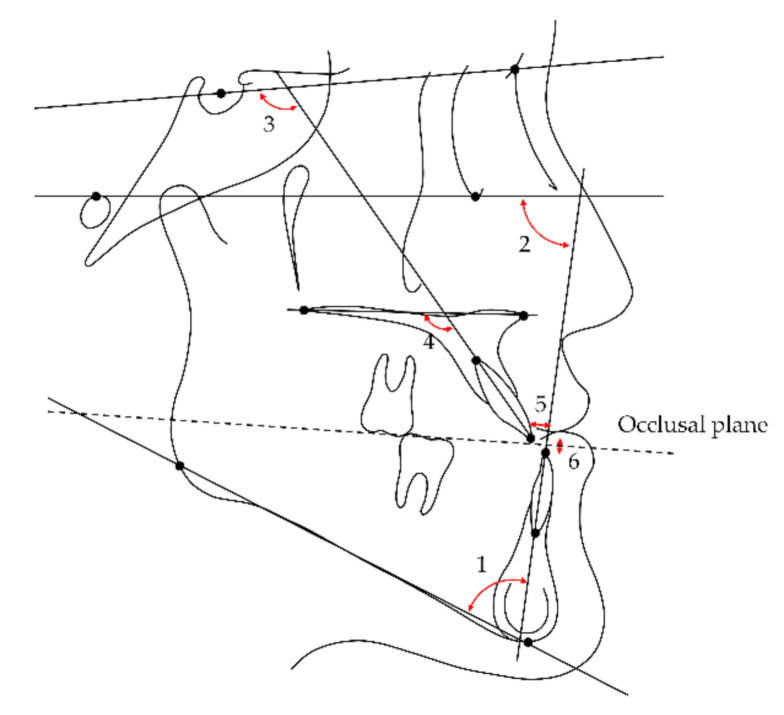
Dental cephalometric variables measured in this study (overjet and overbite were linear measurements whereas the other variables were angular measurements): 1, incisor mandibular plane angle (IMPA); 2, Frankfort-mandibular incisor angle (FMIA); 3, maxillary incisor to SN plane angle (U1 to SN); 4, maxillary incisor to palatal plane angle (U1 to PP); 5, overjet; 6, overbite.

A series of lateral cephalograms were digitized and analyzed using V-Ceph 8.0 software (Osstem Implant, Seoul, Korea) by an investigator blinded to participant information. Landmarks, reference plane, measured cephalometric variables, and their abbreviations used in this study are presented in Figure 1, Figure 2 and Figure 3. Cephalograms obtained less than 3 months postsurgery without a surgical wafer were used as postsurgical images to exclude the effects of surgical wafers on rotation of the mandible. Differences between stages were calculated by subtracting the values of the previous time point from those of the subsequent time point.

### 2.3. Statistical Analysis

In total, 35 cephalograms were randomly selected and cephalometric measurements were repeated at 4-week intervals by the same investigator. The measurement error calculated using Dahlberg’s formula [19] was 0.12 mm for linear measurements and ranged from 0.15–0.51° for angular measurements. Intraclass correlation coefficients (ICCs) were calculated to assess intra-examiner reliability. ICCs exceeded 0.988 for all measurements.

Statistical analysis was performed for all variables at the five stages and differences between stages. Descriptive statistics are presented as mean ± standard deviation and numbers with percentages. Differences in sex distribution, extraction in the maxillary arch, and genioplasty status between groups were analyzed using the Fisher’ exact test. The Mann–Whitney U test was used to analyze differences in age at pretreatment and time periods between stages. Cephalometric variables of both groups at each stage and time period were compared using multiple linear regression analysis adjusted for age and extraction in the maxillary arch. Spearman correlation analysis was performed to investigate the relationship between anteroposterior skeletal variables and incisal variables. IBM SPSS Statistics 25 (IBM, Chicago, Il) was used for all analyses. The significance level was set to 0.05.

## 3. Results

### 3.1. Demographic Data

Demographic data of patients are presented in Table 1. No significant between-group differences were observed in sex distribution, extraction in the maxillary arch, genioplasty status, or age at pretreatment. In addition, time periods for presurgical orthodontic treatment (T1–T2), postsurgical orthodontic treatment (T3–T4), total treatment (T1–T4), and retention (T4–T5) were not significantly different between the two groups (Table 1).

### 3.2. Dentoskeletal Characteristics at Each Stage

Differences in the cephalometric variables between the two groups at each stage are presented in Table 2 and Table 3. No significant between-group differences were observed in dentoskeletal variables at T1 (Table 2). At T2, mandibular incisors of RB were more labially inclined than those of NB by approximately 9° (incisor mandibular plane angle [IMPA], *p* = 0.003 and Frankfort-mandibular incisor angle [FMIA], *p* = 0.006) (Table 2). After orthognathic surgery, the anteroposterior skeletal relationships (ANB and Frankfort horizontal to AB plane angle [FABA]) of both groups were significantly improved at T3, resulting in a positive postsurgical overjet at T3 in both groups. Compared to RB, NB presented with a larger overjet (*p* = 0.015) and more lingually inclined mandibular incisors (IMPA, *p* = 0.002; FMIA, *p* = 0.006) at T3 despite similar skeletal relationships between the two groups (Table 2).

NB exhibited a more severe skeletal Class III relationship and lingually inclined mandibular incisors compared to RB at T4 (ANB, *p* = 0.013; FABA, *p* = 0.035; IMPA, *p* = 0.015; FMIA, *p* = 0.030) and T5 (ANB, *p* = 0.005; FABA, *p* = 0.019; IMPA, *p* = 0.013; FMIA, *p* = 0.029) (Table 3).

### 3.3. Changes in Dentoskeletal Characteristics

Changes in dentoskeletal characteristics between stages are presented in Table 4. Significant between-group differences were observed in mandibular incisor inclination (IMPA and FMIA, *p* < 0.001) and overjet (*p* < 0.001) during presurgical orthodontic treatment (T1–T2) (Table 4). No significant between-group differences were observed in dentoskeletal changes during orthognathic surgery (T2–T3) (Table 4), indicating similar surgical changes between groups. However, significant between-group differences were observed in dentoskeletal changes during postsurgical orthodontic treatment (T3–T4). Relapse tendency of the anteroposterior maxillomandibular relationship was greater in NB than in RB during T3–T4 (ANB, *p* < 0.001; FABA, *p* = 0.013) (Table 4). In addition, mandibular incisors were more lingually inclined in RB than in NB (IMPA, *p* = 0.016; FMIA, *p* = 0.022), which contributed to a significant difference in overjet change (*p* = 0.005) between the two groups during T3–T4 (Table 4). No significant between-group differences were noted in dentoskeletal changes during T4–T5 (Table 4).

### 3.4. Relationships between Incisal and Postsurgical Skeletal Variables

IMPA and FMIA at T2 were significantly associated with FABAs at T3, T4, and T5. Lingual inclination of mandibular incisors at T2 was significantly associated with an increase in FABA at T3 (*p* < 0.05), T4 (*p* < 0.01), and T5 (*p* < 0.01). Increased overjet at T3 was significantly associated with decreased ANB and increased FABA during T3–T4 (*p* < 0.05) (Table 5). No significant associations were identified between incisal variables and postsurgical vertical skeletal variables (data not shown).

## 4. Discussion

Dentoalveolar compensation is an adaption of dental components to skeletal discrepancy that manifests in all three spatial planes but is most apparent in the anteroposterior dimension [1]. Retroclined mandibular incisors are one of the most common anteroposterior dentoalveolar compensations in patients with skeletal Class III malocclusion [20], which understates negative overjet. Given that the anteroposterior position of mandibular incisors determines the position of the mandible relative to the maxilla at surgery, it is necessary to eliminate compensation of mandibular incisor inclination prior to surgery [4,12,13]. Presurgical decompensation of mandibular incisor inclination reveals true skeletal discrepancy and enables the desired amount of surgery and stable postsurgical occlusion, ultimately contributing to long-term stability of final Class III surgical orthodontic treatment outcomes [5,7,8,9]. Nevertheless, the relationship between presurgical decompensation of mandibular incisor inclination and stability of Class III surgical orthodontic treatment has not been examined to date. The purpose of this study was therefore to investigate the effect of mandibular incisor decompensation on long-term outcomes of surgical orthodontic treatment of skeletal Class III malocclusion.

Despite the similar pretreatment conditions between the two groups (Table 1 and Table 2), mandibular incisor inclination in NB was maintained during presurgical orthodontic treatment (T1–T2), whereas in the RB group, significant labial inclination of the mandibular incisors was observed (Table 4). These results suggest that the dentoalveolar compensation of the mandibular incisors was successfully eliminated only in the RB group (Table 2) due to the different settings of inclination of the mandibular incisors according to the bracket placement method. The differences in changes in mandibular incisor inclination may have underpinned differences in overjet changes between the two groups during T1–T2 (Table 4). However, there was no significant difference in overjet at T2, which may be associated with slightly more labially inclined maxillary incisors (U1 to SN and U1 to PP) and slightly larger negative overjet in NB than in RB at T1 (Table 2).

Although the overjet in both groups was significantly changed by approximately 8.5 mm at postsurgery (Table 4), overjet at T3 was larger in NB than in RB (4.32 ± 1.31 and 3.21 ± 0.98, respectively; Table 2). However, skeletal variables (ANB and FABA) at both T2 and T3 were not significantly different between the two groups (Table 2). A potential reason for this is that molar relationships, in addition to interincisal relationships, are set to achieve a harmonious jaw–face relationship when determining postsurgical occlusion [21,22]. Indeed, the formation of a harmonious jaw-face relationship via establishment of a desirable intermaxillary relationship is just as important as a good occlusal relationship. This may have underpinned the lack of significant differences in skeletal changes during surgery between the two groups in this study (Table 4).

Although no significant differences were observed in ANB and FABA at T3, both groups demonstrated decreased ANB and increased FABA during postsurgical orthodontic treatment (T3–T4) (Table 4), indicating anteroposterior skeletal relapse tendency after orthognathic surgery. Relapse tendency may significantly influence changes in overjet and mandibular incisor inclination during T3–T4. As NB had a larger overjet (looser interincisal contact) at postsurgery compared to RB (Table 2), skeletal relapse tendency reduced overjet without significant changes in mandibular incisor inclination (IMPA and FMIA) in NB (Table 4). In contrast, skeletal relapse tendency may have induced lingual inclination of the mandibular incisors without significant overjet changes in RB (Table 4) due to a smaller overjet and tighter interincisal contact at T3 (Table 2). Notably, despite postsurgical lingual inclination of the mandibular incisors, RB still exhibited more labially inclined mandibular incisors (IMPA and FMIA) by approximately 6° compared to NB at T4 and T5 (Table 3). This could be due to the successful elimination of dentoalveolar compensation of mandibular incisor inclination (IMPA and FMIA) in RB during presurgical orthodontic treatment (Table 4). These results suggest that presurgical decompensation of mandibular incisor inclination may persist over time.

**Figure 4:**
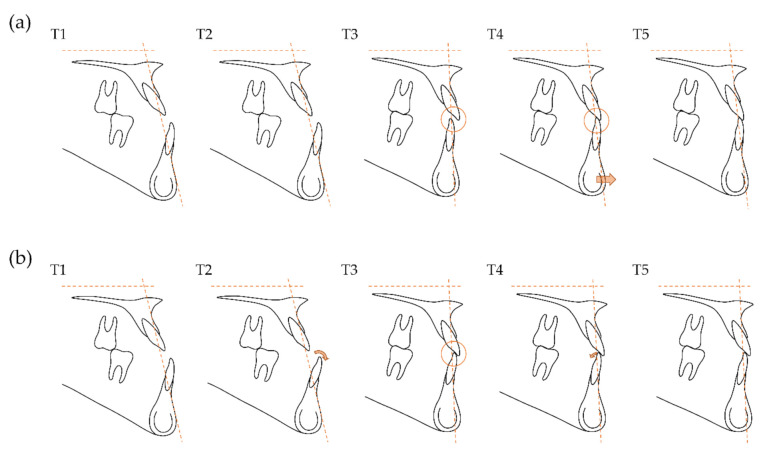
Schematic diagram of dentoskeletal changes of normal bracketing group (NB) (**a**) and reverse bracketing group (RB) (**b**). The horizontal dashed line represents the Frankfort horizontal plane and the oblique vertical dashed line represents the line connecting points A and B. Arrows indicate the difference in dentoskeletal changes between the two groups. Although both groups undergone similar skeletal changes during orthognathic surgery, the decompensation of the mandibular incisor inclination during presurgical orthodontic treatment was insufficient in NB, resulting in a larger overjet and looser interincisal contact compared to RB at postsurgery. Both groups experienced anteroposterior relapse during postsurgical orthodontic treatment, but NB had more anteroposterior skeletal relapse than RB without significant change in mandibular incisor inclination change: T1, pretreatment; T2, presurgery; T3, postsurgery; T4, posttreatment; T5, retention.

Although both groups demonstrated skeletal relapse tendency after surgery, NB exhibited a more severe skeletal Class III relationship (ANB and FABA) compared to RB at T4 and T5 (Table 3), indicating greater postsurgical anteroposterior skeletal relapse in NB than in RB despite similar surgical changes in both groups (Table 4). The only incisal variables that differed significantly between the two groups at T2 and T3 were the IMPA and FMIA at T2 and T3 and overjet at T3 (Table 2). Correlation analysis of incisal variables and postsurgical skeletal variables revealed that FABAs increased at T3, T4, and T5 with greater lingual inclination of mandibular incisors (IMPA and FMIA) at T2 (Table 5). In addition, a larger overjet at T3 was significantly associated with decreased ANB and increased FABA during postsurgical orthodontic treatment (T3–T4) (Table 5). These findings indicate that presurgical mandibular incisor inclination and postsurgical overjet were significantly associated with skeletal Class III maxillomandibular relationships at postsurgical stages. In this regard, postsurgical relapse may be due to unstable postsurgical occlusion with a lack of interincisal contact [11,23]. Proper decompensation of mandibular incisor inclination at presurgery may enable a smaller overjet with tighter interincisal contact at postsurgery and facilitate the maintenance of stable postsurgical occlusion thereafter (Figure 4). In contrast, insufficient decompensation of mandibular incisor inclination may result in a larger overjet with a more deficient interincisal contact at postsurgery, which may promote skeletal relapse tendency and adversely affect long-term outcomes of Class III surgical orthodontic treatment (Figure 4). In addition, compensation of deficient interincisal contact with Class II mechanics may partly enhance anteroposterior skeletal relapse during postsurgical orthodontic treatment. These results suggest that such skeletal relapse tendencies should be considered when establishing a treatment plan [9,10,23], especially when applying minimal presurgical orthodontic treatment [10] or surgery-first [11] approaches that perform orthognathic surgery without sufficient decompensation of mandibular incisor inclination.

Both groups exhibited minimal dentoskeletal changes during retention, and no significant between-group differences were noted in this regard (Table 4). The majority of postsurgical changes including dentoskeletal relapse occurred during postsurgical orthodontic treatment (T3–T4), in accordance with a previous study reporting that most skeletal relapse occurs within the first 6 months [6]. Based on the greater skeletal relapse tendency in NB than in RB during T3–T4, presurgical mandibular incisor decompensation may more strongly influence dentoskeletal changes during postsurgical orthodontic treatment but less so during retention.

No specific side effects related to periodontal tissues were observed in patients in this study because reverse bracketing was applied in patients with sufficient alveolar housing. However, gingival recession and dehiscence will occur if incisor roots are moved out of alveolar housing due to excessive decompensation [24]. In particular, the vertical alveolar bone level, alveolar bone thickness, and width of attached gingiva may be reduced in the mandibular incisor area after decompensation of mandibular incisor inclination [18,25]. Therefore, careful examination of soft tissue condition and alveolar bone thickness before treatment is required, and excessive proclination of the mandibular incisors should be avoided.

The results of this study suggest that the establishment of proper mandibular incisor inclination by sufficient presurgical mandibular incisor decompensation and formation of adequate postsurgical overjet may contribute to long-term stability of conventional surgical orthodontic treatment. Mandibular incisor decompensation by reverse bracket positioning is more convenient than provision of a third order bend with an archwire and/or using Class II elastics. In addition, methods such as forward movement of the mandibular dentition using orthodontic mini-implants or miniplates may be applied for additional decompensation of mandibular incisor inclination.

For cases of thin mandibular alveolar housing, complete elimination of mandibular incisor compensation is not possible; therefore, different strategies may be considered. The deployment of mandibular incisor brackets with approximately 0° of inclination to avoid excessive mandibular incisor decompensation or establishment of proper postsurgical interincisal contact by avoiding excessive retroclination of the maxillary incisors are possible orthodontic options for presurgical orthodontic treatment. In addition, establishment of proper postsurgical inclination of the maxillary incisors is required and can be achieved by controlling the rotation of the maxilla during surgery. If the aforementioned strategies prove inadequate, overcorrection of the anteroposterior skeletal relationship through surgery may be considered as an alternative.

This study has several limitations including its retrospective design and the use of lateral cephalograms, which only provide a two-dimensional view. To further clarify the effects of presurgical mandibular incisor decompensation on the relationship between postsurgical overjet and long-term treatment stability, prospective studies using three-dimensional images such as computed tomography are warranted. Esthetic improvement of soft tissue is one of the main reasons for seeking surgical orthodontic treatment [26]; however, this study only focused on dentoskeletal changes. Further studies are needed to investigate the long-term relationships between mandibular incisor decompensation and soft tissue changes. In addition, the relationship between the mandibular incisor inclination and alveolar bone remodeling should be further investigated, especially in patients with thin alveolar housing, to develop criteria for determining the limits of the mandibular incisor decompensation without periodontal side effects.

## 5. Conclusions

In conclusion, this study analyzed the effects of presurgical mandibular incisor decompensation on long-term outcomes of skeletal Class III surgical orthodontic treatment. Although no significant differences were identified in pretreatment dentoskeletal characteristics and surgical changes between the two groups, NB demonstrated a larger overjet at postsurgery and a more severe skeletal Class III relationship with a smaller ANB and larger FABA at posttreatment and retention compared to RB. Correlation analysis revealed that a greater lingual inclination of the mandibular incisors at presurgery and larger overjet at postsurgery were significantly associated with skeletal relapse toward more severe Class III relationships during postsurgical orthodontic treatment. These findings suggest that the establishment of adequate postsurgical overjet by sufficient presurgical mandibular incisor decompensation may contribute to long-term stability of Class III surgical orthodontic treatment.

## Figures and Tables

**Table 1:** Demographic data of patients.

Demographics	Normal Bracketing	Reverse Bracketing	Significance
Patients (% of total)	18 (51.4)	17 (48.6)	
Sex (% of group)			
Male	12 (66.7)	7 (41.2)	0.181 ^1^
Female	6 (33.3)	10 (58.8)	
Extraction in the maxillary arch (% of group)			
Extraction	7 (38.9)	4 (23.5)	0.471 ^1^
Non-extraction	11 (61.1)	13 (76.5)	
Genioplasty (% of group)			
Yes	7 (38.9)	10 (58.8)	0.318 ^1^
No	11 (61.1)	7 (41.2)	
Age at pretreatment (years)			
Mean	20.76 ± 2.99	21.36 ± 3.63	0.779 ^2^
Range	17.25–29.67	17.33–28.33	
Time periods between stages (months)			
Presurgical orthodontic treatment (T–T2)	10.67 ± 3.01	11.88 ± 4.59	0.590 ^2^	
Postsurgical orthodontic treatment (T3–T4)	6.83 ± 2.92	6.88 ± 2.29	0.946 ^2^
Total treatment (T1–T4)	19.39 ± 5.27	20.35 ± 5.62	0.590 ^2^
Retention (T4–T5)	10.78 ± 7.17	9.76 ± 5.70	0.868 ^2^

Normal bracketing: Mandibular incisor brackets with −6° of inclination were placed normally. Reverse bracketing: Mandibular incisor brackets with −6° of inclination were placed inversely (+6° of inclination). T1: pretreatment; T2: presurgery; T3: postsurgery; T4: posttreatment; T5: retention. ^1^ The Fisher’s exact test was used to analyze the significance of differences between the two groups. ^2^ The Mann–Whitney U test was used to analyze the significance of differences between the two groups.

**Table 2:** Differences in dentoskeletal characteristics between the two groups at pretreatment, presurgery, and postsurgery.

Variables	Pretreatment (T1)			Presurgery (T2)			Postsurgery (T3)		
	Normal Bracketing	Reverse Bracketing	Sig ^1^	Normal Bracketing	Reverse Bracketing	Sig ^1^	Normal Bracketing	Reverse Bracketing	Sig ^1^
FMA	26.30 ± 6.48	26.53 ± 5.64	0.948	26.77 ± 6.43	26.84 ± 5.62	0.997	28.88 ± 5.9	27.43 ± 4.33	0.327
SN-MP	36.23 ± 7.06	36.74 ± 5.91	0.836	36.88 ± 6.71	37.00 ± 6.08	0.953	39.19 ± 5.73	37.98 ± 6.33	0.472
SNA	81.74 ± 3.64	80.95 ± 3.78	0.493	81.54 ± 3.42	81.31 ± 3.65	0.742	82.60 ± 2.58	82.39 ± 4.44	0.837
SNB	84.63 ± 4.35	83.18 ± 3.95	0.372	84.16 ± 4.09	83.29 ± 4.09	0.595	79.70 ± 3.03	78.95 ± 4.55	0.559
ANB	−2.89 ± 2.65	−2.23 ± 1.64	0.585	−2.62 ± 2.77	−1.98 ± 1.88	0.661	2.90 ± 1.52	3.44 ± 1.87	0.384
FABA	98.95 ± 6.79	96.72 ± 3.85	0.396	98.25 ± 6.83	96.37 ± 4.52	0.545	85.3 ± 4.11	83.76 ± 4.85	0.339
IMPA	83.63 ± 7.14	81.91 ± 6.76	0.566	83.23 ± 8.82	92.01 ± 5.91	0.003	82.07 ± 8.27	91.36 ± 7.35	0.002
FMIA	70.07 ± 6.66	71.56 ± 7.11	0.599	70.00 ± 9.30	61.15 ± 7.14	0.006	69.05 ± 7.91	61.2 ± 6.78	0.006
U1 to SN	116.57 ± 5.34	112.36 ± 6.44	0.086	111.04 ± 9.43	108.79 ± 8.08	0.398	107.05 ± 7.63	105.00 ± 7.02	0.420
U1 to PP	126.14 ± 4.70	123.41 ± 5.19	0.231	120.68 ± 8.75	119.77 ± 7.33	0.670	120.94 ± 8.29	119.72 ± 7.56	0.571
Overjet	−2.68 ± 2.61	−1.25 ± 2.72	0.178	−4.21 ± 3.46	−5.36 ± 3.01	0.100	4.32 ± 1.31	3.21 ± 0.98	0.015
Overbite	0.39 ± 2.36	−0.19 ± 2.56	0.483	0.51 ± 1.68	−0.40 ± 2.08	0.223	1.75 ± 0.55	1.27 ± 1.13	0.129

Normal bracketing: Mandibular incisor brackets with −6° of inclination were placed normally. Reverse bracketing: Mandibular incisor brackets with −6° of inclination were placed inversely (+6° of inclination). FMA, Frankfort-mandibular plane angle; SN-MP, sella-nasion to mandibular plane angle; FABA, Frankfort horizontal to AB plane angle; IMPA, incisor mandibular plane angle; FMIA, Frankfort-mandibular incisor angle; U1 to SN, maxillary incisor to SN plane angle; U1 to PP, maxillary incisor to palatal plane angle. The units for all variables are in degrees, with the exception of overjet and overbite which are presented in millimeters. ^1^ Significance: Multiple linear regression analysis adjusted for age and extraction in the maxillary arch was performed.

**Table 3:** Differences in dentoskeletal characteristics between the two groups at posttreatment and retention.

Variables	Posttreatment (T4)			Retention (T5)		
	Normal Bracketing	Reverse Bracketing	Sig ^1^	Normal Bracketing	Reverse Bracketing	Sig ^1^
FMA (°)	29.51 ± 5.83	28.27 ± 4.32	0.385	29.46 ± 6.24	28.20 ± 4.03	0.405
SN-MP (°)	39.65 ± 6.05	38.71 ± 5.72	0.555	39.56 ± 6.45	38.73 ± 5.63	0.611
SNA (°)	81.97 ± 2.95	82.26 ± 4.46	0.823	81.82 ± 3.04	82.16 ± 4.73	0.781
SNB (°)	80.53 ± 3.29	79.43 ± 4.47	0.413	80.85 ± 3.58	79.66 ± 4.73	0.416
ANB (°)	1.45 ± 1.49	2.83 ± 1.61	0.013	0.97 ± 1.43	2.49 ± 1.59	0.005
FABA (°)	88.21 ± 3.89	85.34 ± 3.59	0.035	89.28 ± 3.71	86.05 ± 3.88	0.019
IMPA (°)	81.27 ± 7.13	88.24 ± 7.95	0.015	80.91 ± 6.89	87.72 ± 7.85	0.013
FMIA (°)	69.22 ± 7.20	63.49 ± 6.33	0.030	69.64 ± 6.72	64.07 ± 6.53	0.029
U1 to SN (°)	109.29 ± 6.85	108.42 ± 5.67	0.679	109.58 ± 7.24	108.05 ± 6.62	0.444
U1 to PP (°)	122.93 ± 7.44	122.94 ± 6.3	0.874	123.25 ± 7.74	122.5 ± 6.51	0.578
Overjet (mm)	3.61 ± 0.90	3.61 ± 0.91	0.971	3.38 ±1.00	3.37 ± 0.92	0.861
Overbite (mm)	1.95 ± 0.50	1.72 ± 0.69	0.327	1.90 ± 1.00	1.62 ± 0.96	0.479

Normal bracketing: Mandibular incisor brackets with −6° of inclination were placed normally. Reverse bracketing: Mandibular incisor brackets with −6° of inclination were placed inversely (+6° of inclination). FMA, Frankfort-mandibular plane angle; SN-MP, sella-nasion to mandibular plane angle; FABA, Frankfort horizontal to AB plane angle; IMPA, incisor mandibular plane angle; FMIA, Frankfort-mandibular incisor angle; U1 to SN, maxillary incisor to SN plane angle; U1 to PP, maxillary incisor to palatal plane angle. ^1^ Significance: Multiple linear regression analysis adjusted for age and extraction in the maxillary arch was performed.

**Table 4:** Changes in dentoskeletal characteristics of the two groups at each time period.

Variables	Presurgical Orthodontics (T1–T2)			Orthognathic Surgery (T2–T3)			Postsurgical Orthodontics (T3–T4)			Retention (T4–T5)		
	Normal Bracketing	Reverse Bracketing	Sig ^1^	Normal Bracketing	Reverse Bracketing	Sig ^1^	Normal Bracketing	Reverse Bracketing	Sig ^1^	Normal Bracketing	Reverse Bracketing	Sig ^1^
FMA (°)	0.47 ± 1.08	0.32 ± 1.09	0.727	2.11 ± 4.32	0.59 ± 4.67	0.259	0.63 ± 1.26	0.84 ± 2.04	0.777	−0.06 ± 1.14	−0.07 ± 1.23	0.967
SN-MP (°)	0.65 ± 0.95	0.25 ± 1.46	0.423	2.3 ± 4.28	0.98 ± 4.66	0.303	0.46 ± 1.05	0.74 ± 1.60	0.575	−0.09 ± 0.92	0.01 ± 1.26	0.741
SNA (°)	−0.20 ± 1.09	0.36 ± 1.66	0.321	1.06 ± 2.09	1.08 ± 1.95	0.848	−0.62 ± 1.09	−0.13 ± 0.72	0.095	−0.15 ± 1.02	−0.10 ± 0.62	0.769
SNB (°)	−0.47 ± 0.90	0.11 ± 1.68	0.243	−4.46 ± 2.53	−4.34 ± 2.17	0.975	0.83 ± 0.94	0.48 ± 0.80	0.299	0.32 ± 1.02	0.23 ± 0.68	0.818
ANB (°)	0.27 ± 0.46	0.25 ± 0.76	0.784	5.52 ± 2.60	5.42 ± 1.28	0.814	−1.46 ± 0.54	−0.61 ± 0.43	< 0.001	−0.47 ± 0.42	−0.34 ± 0.36	0.239
FABA (°)	−0.70 ± 1.26	−0.35 ± 1.96	0.468	−12.95 ± 6.12	−12.62 ± 3.99	0.872	2.91 ± 1.22	1.58 ± 1.63	0.013	1.07 ± 1.30	0.71 ± 1.21	0.379
IMPA (°)	−0.40 ± 6.73	10.10 ± 5.38	< 0.001	−1.16 ± 3.62	−0.64 ± 4.63	0.566	−0.80 ± 2.93	−3.13 ± 2.08	0.016	−0.36 ± 2.1	−0.51 ± 1.49	0.988
FMIA (°)	−0.07 ± 6.46	−10.41 ± 5.37	< 0.001	−0.95 ± 4.30	0.05 ± 5.22	0.577	0.16 ± 2.93	2.29 ± 2.06	0.022	0.42 ± 1.97	0.58 ± 1.32	0.988
U1 to SN (°)	−5.53 ± 7.42	−3.57 ± 8.49	0.772	−3.98 ± 4.19	−3.79 ± 4.46	0.757	2.24 ± 2.16	3.42 ± 4.57	0.357	0.29 ± 3.00	−0.37 ± 2.15	0.302
U1 to PP (°)	−5.46 ± 7.74	−3.63 ± 8.36	0.811	0.26 ± 3.33	−0.05 ± 3.87	0.761	1.99 ± 2.65	3.22 ± 4.46	0.354	0.32 ± 3.28	−0.43 ± 1.99	0.296
Overjet (mm)	−1.53 ± 1.93	−4.11 ± 2.73	< 0.001	8.53 ± 3.72	8.56 ± 2.80	0.497	−0.71 ± 1.32	0.41 ± 0.44	0.005	−0.23 ± 0.67	−0.24 ± 0.43	0.808
Overbite (mm)	0.12 ± 1.54	−0.20 ± 2.71	0.881	1.23 ± 1.70	1.67 ± 1.71	0.648	0.20 ± 0.70	0.45 ± 0.78	0.317	−0.05 ± 0.73	−0.11 ± 0.43	0.879

Normal bracketing: Mandibular incisor brackets with −6° of inclination were placed normally. Reverse bracketing: Mandibular incisor brackets with −6° of inclination were placed inversely (+6° of inclination). T1: pretreatment; T2: presurgery; T3: postsurgery; T4: posttreatment; T5: retention. FMA, Frankfort-mandibular plane angle; SN-MP, sella-nasion to mandibular plane angle; FABA, Frankfort horizontal to AB plane angle; IMPA, incisor mandibular plane angle; FMIA, Frankfort-mandibular incisor angle; U1 to SN, maxillary incisor to SN plane angle; U1 to PP, maxillary incisor to palatal plane angle. ^1^ Significance: Multiple linear regression analysis adjusted for age and extraction in the maxillary arch was performed.

**Table 5:** Relationships between incisal and postsurgical skeletal variables.

Postsurgical Skeletal Variables	Incisal Variables					
	IMPA at Presurgery (T2)		FMIA at Presurgery (T2)		Overjet at Postsurgery (T3)	
	Corr	Sig	Corr	Sig	Corr	Sig
ANB						
Postsurgery (T3)	0.136	0.437	−0.125	0.473	0.070	0.688
Posttreatment (T4)	0.206	0.235	−0.183	0.292	−0.075	0.670
Retention (T5)	0.189	0.277	−0.148	0.396	−0.068	0.698
Change in postsurgical orthodontic treatment (T3–T4)	0.021	0.905	−0.107	0.541	−0.428 *	0.010
Change in retention (T4–T5)	−0.029	0.869	0.093	0.597	−0.224	0.197
FABA						
Postsurgery (T3)	−0.376 *	0.026	0.423 *	0.011	−0.100	0.569
Posttreatment (T4)	−0.437 **	0.009	0.521 **	0.001	0.019	0.913
Retention (T5)	−0.440 **	0.008	0.507 **	0.002	0.072	0.679
Change in postsurgical orthodontic treatment (T3-T4)	0.038	0.830	0.143	0.414	0.406 *	0.016
Change in retention (T4-T5)	−0.008	0.962	−0.020	0.911	0.189	0.278

Corr, Spearman correlation; Sig, Significance; * *p* < 0.05; ** *p* < 0.01. IMPA, incisor mandibular plane angle; FMIA, Frankfort-mandibular incisor angle; FABA, Frankfort horizontal to AB plane angle.
